# Presence and distribution of the lubricating protein, lubricin, in the meibomian gland in rabbits

**Published:** 2011-11-23

**Authors:** Thomas Cheriyan, Thomas M. Schmid, Myron Spector

**Affiliations:** 1 Tissue Engineering, VA Boston Healthcare System, Boston, MA; 2 Department of Orthopedic Surgery, Brigham and Women’s Hospital, Harvard Medical School, Boston, MA; 3 Department of Biochemistry, Rush University Medical Center, Chicago, IL

## Abstract

**Purpose:**

Lubricin is a principal boundary lubricating and anti-adhesion protein found in synovial fluid and several musculoskeletal tissues. This study investigates the presence of lubricin in the meibomian gland, lacrimal gland and ocular surface of healthy rabbits; prompted by the hypothesis that lubricin acts as boundary lubricant and anti-adhesive protein in the eye.

**Methods:**

Thirty six eyelids were resected from ten cadaveric New Zealand White rabbits and two eyeballs and two lacrimal glands from two of them. Thirty two samples from 8 animals were processed for immunohistochemical localization of lubricin using a purified monoclonal antibody and quantification of the lubricin-containing meibocytes. Confirmatory western blot analysis was performed on four eyelids from 2 animals.

**Results:**

Lubricin-positive meibomian cells were seen in the glands in all eight animals evaluated immunohistochemically. The percentage of lubricin-positive cells ranged from was 8%–50% in the upper and 3%–50% in the lower eyelid, with no significant difference between the upper and lower eyelid. Western blot analysis confirmed the presence of lubricin ranging from 10 to 40 ng in four eyelids from the other two rabbits. Occasional staining was seen in the epithelium of the hair follicles of the eyelid. No lubricin was evident on the ocular surface or in the lacrimal gland.

**Conclusions:**

Lubricin is secreted by the meibomian gland. The results provide a basis for the hypothesis that lubricin plays a role in boundary lubrication and in preventing adhesions in the eye, as well as in contributing to other functions of the meibomian gland. Moreover, if lubricin functions to decrease the friction between the eyelid and ocular surface, this study provides a rationale to supplement the amount of lubricin in cases of compromised meibomian gland function and other conditions.

## Introduction

The meibomian glands, also known as tarsal glands, are modified sebaceous glands located within the tarsal plates of both the upper and lower eyelid. The secretions of these holocrine glands, along with dead cell remnants, are released into the lumen by rupture of the plasma membrane. The secretion of the gland acini, meibum, enters into the ductule. Ductules join together to form the central duct which opens into the lid margin at the mucocutaneous junction of the eyelid. During blinking, contraction of the orbicularis oculi and muscle of Riolan results in meibum being released onto the posterior lid margin [1-3]. Neuronal and hormonal control of the gland has previously been demonstrated [4,5].

Meibum contributes to the lipid bilayer of the tear film situated at the air-tear film interface. This layer comprises non polar lipids (wax esters and steroid esters) and lesser amounts of polar lipids [6]. The inner layer of the bilayer, adjacent to the aqueous layer, comprises polar lipids and the outer layer, at the air-tear film interface, comprises non polar lipids [7]. The secretion of the meibomian gland functions to: maintain stability of the tear film; help prevent tear evaporation; prevent contamination of the tear film; and lower surface tension [8,9].

The ocular tear film is essential for lubrication between the inner surface of the eyelids and cornea, and it also provides an optically smooth surface [7]. The tear film has three layers: the innermost mucous layer secreted by the adjacent corneal epithelium; an intermediate aqueous layer produced by the lacrimal gland; an outermost lipid layer produced by the meibomian gland [10-12]. During blinking, frictional forces can be rationalized to be highest at the outermost lipid bilayer of the tear film, prompting the search for a boundary lubricating molecule synthesized by the meibomian gland. That the mucinous glycoprotein, lubricin, has been identified in recent years as the principal lubricating protein in the body focused attention on its synthesis by meibomian cells.

Lubricin, which has been shown to play a vital role in the lubrication of joints [13,14], was first isolated from bovine synovial fluid by Swann, et al., in 1982 [15], and found to be synthesized by synovial cells [16]. Later work by Schumacher, et al. [17],  described a proteoglycan, superficial zone protein (SZP), produced by the chondrocytes in superficial zone of bovine articular cartilage [17]. Subsequent studies showed that SZP was homologous to megakaryocyte stimulating factor [18] and to lubricin [19], and that these homologous glycoproteins were encoded by 12 exons of the proteoglycan 4 (*Prg4*) gene [20]. Immunohistochemical localization of lubricin has since demonstrated its presence in tendon [21], meniscus [22], ligament [23], muscle [23], skin [23], and intervertebral disc [24].

Lubricin has a molecular weight of around 2×10^5^ Da (by sedimentation analysis), intrinsic viscosity of 92 ml/g, and a diffusion coefficient of 1.10×10^−7^ cm^2^/s (light-scattering measurements) [15]. The molecule is an alternately spliced 1,404 amino acid protein, having a NH_2_-terminal somatomedin B (SMB)-like domain and a COOH-terminal hemopexin (PEX)-like domain, joined together by a central mucin-like domain having heavy O-linked glycosylation with NeuAc (2,3)-Gal(1,3)- GalNAc [25,26]. Strong repulsive forces through hydration and steric forces generated by the high negative charge and hydrated sugars in the central domain contribute to lubricin’s lubricating property [25,27,28]. Studies have further shown the amphiphilic and strong absorbent nature of the molecule. On hydrophobic surfaces the molecule adopts a compact loop-like conformation to adsorb anywhere on its hydrophobic domain and on hydrophilic surfaces it adopts a tail like conformation to adsorb anywhere on the hydrophilic central domain [29].

In vitro studies have shown that lubricin in saline buffer acts as a lubricant between various surfaces [30-34], as well as in synovial fluid, providing evidence that lubricin is a principal lubricating protein in joints. Besides the lubricating property, lubricin has been shown to exert anti-adhesive [21] action, strain energy dissipation, and a protective effect on underlying cells [27]. We hypothesized that lubricin is also present in the eye, and therefore may play an important role in the tribology of the eye.

## Methods

Cadaveric tissues from ten New Zealand White rabbits (4–4.5 kg) of either sex were used in this study: 18 upper eyelids; 18 lower eyelids; 2 eyeballs; and 2 lacrimal glands. The rabbits (Millbrook breeding laboratories, Amherst, MA) were euthanized using intravenous phenobarbital (120 mg/kg). None of the animals had any obvious eye pathology at the time of sacrifice, as the animals were part of an institution IACUC approved study involving lower limb surgery. The excised tissues were immediately fixed in 10% buffered formalin for at least 3 days. Normal rabbit articular cartilage was used as controls for lubricin immunohistochemical staining. The myocytes of the orbicularis oculi and squamous epithelium lining the ocular surface of the eyelid served as internal controls.

The length of the upper and lower eyelid margin was measured from the medial to lateral canthus of the eye. Each eyelid was cut in the sagittal plane into 3 sections: a central 4-mm wide zone; a nasal zone; and a temporal zone. All of the specimens were embedded in paraffin blocks after dehydration in graded alcohol solutions and xylene in a tissue processor. Six-μm thick sections were cut along the sagittal plane of the eyelid using a microtome (Shandon Finesse Model ME+; Thermo Fisher Scientific, Waltham, MA). Microtomed sections were fixed to positively charged slides and kept on a slide warmer at 60 °C for at least 2 h. Hematoxylin and eosin staining was done for cell morphology and identification.

Immunohistochemical staining for lubricin was conducted using an autostainer (Universal Staining System; Dako, Carpinteria, CA). The following steps were performed in the autostaining process, separated by rinses with Tris-buffered saline solution containing Tween (TBS, S3006; Dako). To enable antibody penetration, samples were treated with 0.1% protease (type XIV, P5147; Sigma, St. Louis, MO) for 40 min. Endogenous peroxidase activity was quenched by treating the slides with 3% hydrogen peroxide for 10 min and protein block serum-free (X0909; Dako) was used to block nonspecific binding. The slides were then incubated with a primary anti-lubricin monoclonal antibody (S6.79; Rush University Medical Center, Chicago, IL) at a dilution of 1:1,000 (1 µg/ml protein concentration) or with negative mouse immunoglobulin-2b (IgG2b) control at the same dilution (X0944; Dako) for 15 min. The S6.79 anti-lubricin monoclonal antibody is an IgG2b immunoglobulin developed in the mouse against human lubricin [35]. It reacts with lubricin molecules in human, bovine, dog, guinea pig, rabbit [35], and goat [21]. The antibody binds to the NH_2_-terminus of lubricin. Unpublished studies (T.M. Schmid) suggest that the binding epitope is located within the region of the protein coded by exon 3. Therefore, the antibody recognizes the four previously identified human splice variants of lubricin, as each of these variants includes the region coded by exon 3 [22,23].

A labeled streptavidin-biotin horseradish peroxidase system was then used with 3-amino-9-ethylcarbazole (AEC) chromogen to localize the presence of lubricin (LSAB2 system; Dako). In this process, a biotinylated secondary antibody was applied for 15 min, followed by a peroxidase-labeled streptavidin treatment for an additional 15 min. Following this, AEC was applied to the sections for 10 min to allow detection of lubricin as indicated by the presence of red chromogen at the antigen site in the slides. Counterstaining was done with Mayer’s hematoxylin and coverslips were applied to the sections with an aqueous mounting medium (Faramount; Dako).

The immunohistochemical slides were viewed at 100× magnification. Digital images were taken using a Microfire camera (model S99809; Meyer Instruments, Houston, TX) mounted on an Olumpus BX51 microscope (Olympus, Tokyo, Japan). Images were photo-merged using Adobe CS3 photoshop software (San Jose, California). A quantitative cell count was performed on the well differentiated secreting meibocytes in the entire meibomian gland in the 4-mm long central zone of the right upper and right lower eyelid using ImageJ software (National Institutes of Health, Bethesda, MD). Meibocytes with lubricin localized intracellularly or on the cell membrane were counted as lubricin-positive cells. If staining was seen only on one side of the cell membrane of adjacent cells, only one of the cells was taken as a lubricin-positive cell. The percentage of lubricin-positive cells in the meibomian gland was calculated. The paired *t*-test was used to determine the significance of correlation of the percentage of cells staining between the upper and lower eyelid. Linear regression was performed on the total number of cells and the percentage of lubricin-containing cells.

Qualitative analysis of staining was done on 4 animals comparing nasal and temporal zones of the same eyelid and corresponding zones of opposite eyelids.

Western blot analysis was used to confirm the presence of lubricin. Human lubricin was used as control. Lubricin was extracted with 5 ml of 4 M guanidinium chloride, 50 mM Tris, 50 µM benzamidine, 100 µM N-ethylmaleimide, 50 µM phenylmethylsulfonyl fluoride, 0.5% Tween-20, and 10 mM EDTA, pH 7.2 buffer overnight with agitation at 4 °C. Extracts were dialyzed against 50 mM Tris, 1 M NaCl, 0.05% Tween-20, 10 mM EDTA, pH 7.5 and clarified by centrifugation for 15 min at 10,000× g. Lubricin was isolated from the extracts by incubation with 25 µl of peanut lectin-sepharose beads (L2301; EY laboratories, San Mateo, CA) and eluted from the beads by boiling in Laemmli sample buffer. Proteins were separated by SDS–PAGE on 5% polyacrylamide gels [36] and transferred to nitrocellulose for western blotting. Blots were blocked with 5% nonfat milk for 30 min, incubated with S6.79 primary antibody [35] at 2 µg/ml for 2 h, washed and incubated with HRP-conjugated goat anti-mouse IgG (#31432; 1/10,000 dilution; Thermo Scientific,) for 1 h. Lubricin bands were visualized on X-ray film using a SuperSignal West Pico chemiluminescent detection system (#34077; Thermo Scientific).

## Results

**Figure 1:**
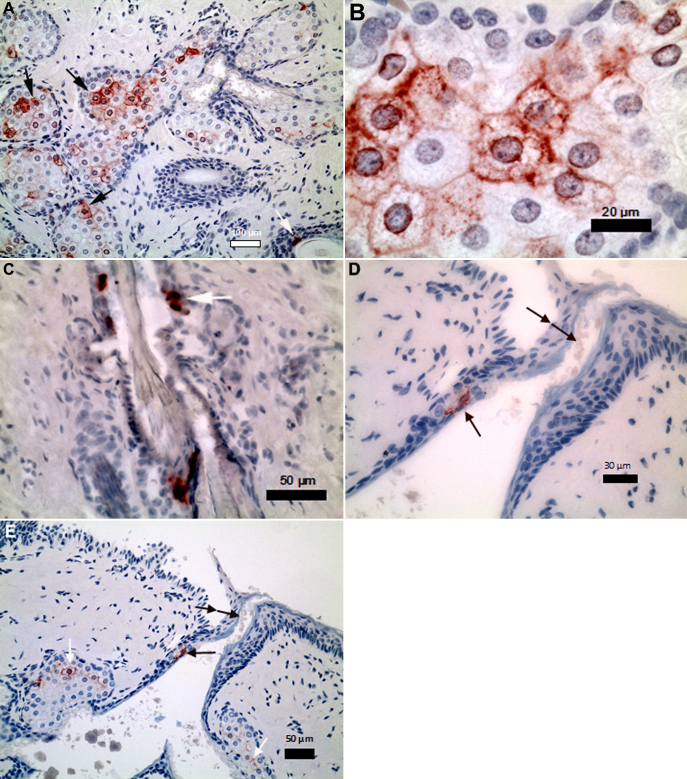
Immunohistochemical micrographs of lubricin (red chromogen). **A**: Low magnification of lubricin-containing meibocytes (black arrows) and lubricin stained hair follicle (white arrow). **B**: High magnification of lubricin-containing meibocytes. **C**: High magnification of a lubricin-stained hair follicle (white arrow) in the eyelid. **D**: High magnification of lubricin staining (black arrow) in the central meibomian duct and equivocal staining of cell debris at the opening (double black arrow). **E**: Low magnification of lubricin staining (black arrow) in the central meibomian duct and equivocal staining of cell debris at the opening (double black arrow).

Histological examination of all eyelids showed normal structures with no obvious pathology. The meibomian gland displayed typical glandular structure distributed in the tarsal muscle. It was observed that the number of well differentiated meibocytes in the upper eyelid was not consistently greater or lesser than that in the lower eyelid. Blood vessels could be seen distributed throughout the sections. All sections of the meibomian gland of every animal showed the presence of lubricin-containing cells (Figure 1A,B). The chromogen was evident intracellularly and on the cell membrane. Occasional intracellular staining was observed in the epithelium of hair follicles in the eyelids (Figure 1A,C). There was no staining for lubricin in corneal, conjunctival, or scleral epithelium and in the lacrimal gland. No lubricin staining was seen on the internal control cells or on any of the immunohistochemical negative control slides stained with mouse IgG2b. The articular cartilage comparative control samples displayed the typical discrete layer of lubricin on the surface of the cartilage.

The ranges of lubricin-containing cells in meibomian gland in the upper and lower eyelids were 8.4%–50.3% and 3.4- 50.3%, respectively (Table 1).

**Table 1:** Percentage of lubricin-containing cells in the meibomian gland.

**Animal #**	**Eyelid**	**Lubricin+ cells**	**Total cell count**	**% Lubricin + cells**
1	Upper	166	521	31.9
	Lower	391	2090	18.8
2	Upper	111	1325	8.4
	Lower	39	1138	3.4
3	Upper	448	945	47.4
	Lower	195	426	45.8
4	Upper	861	2823	30.5
	Lower	199	814	24.4
5	Upper	238	473	50.3
	Lower	234	465	50.3
6	Upper	198	926	21.4
	Lower	352	1450	24.3
7	Upper	219	934	23.4
	Lower	254	1041	24.4
8	Upper	297	955	31.1
	Lower	358	1244	28.8

Linear regression performed on the total number of cells and the percentage of cells staining for lubricin showed no significance. Paired t- test did not reveal a significant difference in the percentage of lubricin-containing cells between the upper and low eyelid in the central zone (p=0.907). Because there was no significant difference in percentage of lubricin-containing cells between the central zones of the upper and lower eyelid, quantitatively, we first did a qualitative comparison between the nasal and temporal zones of the same eyelid and corresponding zones of opposite eyelids. There was no obvious qualitative difference between the zones in the same animal. Thus we did not pursue a quantitative comparison.

A longitudinal section showed chromogen staining in a part of the central meibomian duct (Figure 1D,E). There was equivocal staining of cell debris at the opening of the central duct (Figure 1D,E).

**Figure 2:**
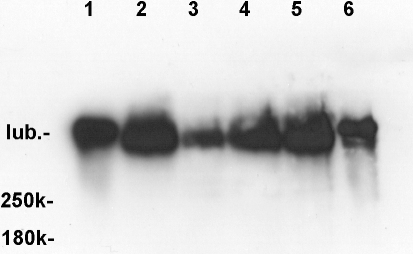
Western blot analysis showing the position of the lubricin bands on the gel relative to the 250K and 180K ProSieve QuadColor protein markers (#00193838; Lonza, Rockland, ME). Lanes 1 and 6 contain 6 ng and 3 ng of human lubricin, respectively. Lanes 2, 3, 4, and 5 are from upper, lower, upper and lower lids from 2 different rabbit eyes.

Western blot analysis confirmed the presence of lubricin. The positions of the lubricin bands on the gel were relative to the 250K and 180K ProSieve QuadColor protein markers (#00193838; Lonza, Rockland, ME; Figure 2). Calibration lanes (1 and 6) in Figure 2 contained 6 ng and 3 ng of human lubricin respectively. Lanes 2, 3, 4, and 5 were from upper, lower, upper and lower lids from 2 different rabbit eyes. One quarter of each sample was run in each lane. Assuming that the lubricin in all rabbit extracts and human lubricin transferred to the nitrocellulose and were detected on the western blots with equivalent efficiencies, then the amount of lubricin extracted was in the range of 10–40 ng from each of the rabbit eyelids.

## Discussion

The results confirmed our hypothesis that lubricin is present in the meibomian gland. All the samples displayed the presence of lubricin-containing meibocytes. That lubricin was identified in meibomian cells would indicate that it is also present in the tear fluid. This supposition is supported by a previous study which found ‘proteoglycan 4; Megakaryocyte stimulating factor’ in one out of seven samples of human meibomian gland secretions, analyzed using a high-performance liquid chromatography mass spectrometry (μHPLC/MS/MS) technique [37]. While the prior work did not identify the protein specifically as lubricin, our work would indicate that it was. The inability of the prior analysis of meibomian gland secretions to identify ‘proteoglycan 4; Megakaryocyte stimulating factor’ consistently in all samples might have been due to the difficulty in collecting and processing meibomian gland secretions without the loss or denaturation of proteins.

The roles of lubricin in the eye might include: lubrication; the prevention of adhesions; surfactant; and/or prevention of evaporative tear loss. The mechanisms involved in the tribology of the eye have long been an enigma to researchers. Numerous models have been proposed to explain the mechanism of lubrication of the eye [38,39]. This is the first study to provide evidence of the likely role of a boundary lubricating protein, lubricin, synthesized by the meibomian gland. In musculoskeletal tissues, lubricin has been found in rats, dogs, and goats, in addition to rabbits and humans. In joints, lubricin functions as a boundary lubricating molecule on the surfaces of articular cartilage and menisci to reduce friction and wear. Lubricin has also been found to serve as an interfascicular lubricant to lower the friction and wear within tendons [21]. It is likely that the molecular properties of lubricin in musculoskeletal tissues, making it a favorable boundary lubricant and anti-adhesive, apply to the eye as well.

The immunohistochemical method used here with paraffin-embedded tissue may not be of use for detecting lubricin in the tear film because of the difficulty of preserving the tear film during processing. Thus other methodological approaches like ELISA will have to be taken to analyze lubricin in the tear secretion.

It was unclear if the lubricin-staining in the central meibomian duct was a result of diffusion or adherence of lubricin from the meibum or if it was staining of meibocytes or ductal epithelial cells. However it provides suggestive evidence that lubricin might prevent adhesion of cells and cell remnants to prevent blockage of ductules and the central duct. Further studies are necessary to understand its role in disease states especially in meibomian gland dysfunction [40] including contact lens induced dysfunction [41].

If lubricin functions to decrease the friction between the eyelid and ocular surface, this study provides a rationale to supplement the amount of lubricin in cases of compromised meibomian gland function.

## References

[1] Bron AJ, Tiffany JM (1998). The meibomian glands and tear film lipids - Structure, function, and control.. Adv Exp Med Biol.

[2] Chew CKS, Hykin PG, Jansweijer C, Dikstein S, Tiffany JM, Bron AJ (1993). The Casual Level of Meibomian Lipids in Humans.. Curr Eye Res.

[3] Korb DR, Baron DF, Herman JP, Finnemore VM, Exford JM, Hermosa JL, Leahy CD, Glonek T, Greiner JV (1994). Tear Film Lipid Layer Thickness as a Function of Blinking.. Cornea.

[4] Seifert P, Spitznas M (1996). Immunocytochemical and ultrastructural evaluation of the distribution of nervous tissue and neuropeptides in the meibomian gland.. Graefes Arch Clin Exp Ophthalmol.

[5] Sullivan DA, Sullivan BD, Ullman MD, Rocha EM, Krenzer KL, Cermak JM, Toda I, Doane MG, Evans JE, Wickham LA (2000). Androgen influence on the meibomian gland.. Invest Ophthalmol Vis Sci.

[6] Butovich IA, Millar TJ, Ham BM (2008). Understanding and analyzing meibomian lipids - A review.. Curr Eye Res.

[7] Holly FJ (1973). Formation and Rupture of Tear Film.. Exp Eye Res.

[8] Tiffany JM (1985). The Role of Meibomian Secretion in the Tears.. Trans Ophthalmol Soc U K.

[9] Driver PJ, Lemp MA (1996). Meibomian gland dysfunction.. Surv Ophthalmol.

[10] Wolff E. Anatomy of eye and orbit. New York: Blakiston; 1954.

[11] Holly FJ, Holly TF (1994). Advances in ocular tribology.. Adv Exp Med Biol.

[12] Corfield AP, Carrington SD, Hicks SJ, Berry M, Ellingham R (1997). Ocular mucins: Purification, metabolism and functions.. Prog Retin Eye Res.

[13] Swann DA, Hendren RB, Radin EL, Sotman SL, Duda EA (1981). The Lubricating Activity of Synovial-Fluid Glycoproteins.. Arthritis Rheum.

[14] Jay GD, Torres JR, Warman ML, Laderer MC, Breuer KS (2007). The role of lubricin in the mechanical behavior of synovial fluid.. Proc Natl Acad Sci USA.

[15] Swann DA, Slayter HS, Silver FH (1981). The Molecular-Structure of Lubricating Glycoprotein-I, the Boundary Lubricant for Articular-Cartilage.. J Biol Chem.

[16] Rhee DK, Marcelino J, Baker MA, Rocha Gong Y, Smits P, Lefebvre V, Jay GD, Stewart M, Wang H, Warman ML, Carpten JD (2005). The secreted glycoprotein lubricin protects cartilage surfaces and inhibits synovial cell overgrowth.. J Clin Invest.

[17] Schumacher BL, Block JA, Schmid TM, Aydelotte MB, Kuettner KE (1994). A Novel Proteoglycan Synthesized and Secreted by Chondrocytes of the Superficial Zone of Articular-Cartilage.. Arch Biochem Biophys.

[18] Flannery CR, Hughes CE, Schumacher BL, Tudor D, Aydelotte MB, Kuettner KE, Caterson B (1999). Articular cartilage superficial zone protein (SZP) is homologous to megakaryocyte stimulating factor precursor and is a multifunctional proteoglycan with potential growth-promoting, cytoprotective, and lubricating properties in cartilage metabolism.. Biochem Biophys Res Commun.

[19] Jay GD, Tantravahi U, Britt DE, Barrach HJ, Cha CJ (2001). Homology of lubricin and superficial zone protein (SZP): products of megakaryocyte stimulating factor (MSF) gene expression by human synovial fibroblasts and articular chondrocytes localized to chromosome 1q25.. J Orthop Res.

[20] Englert C, McGowan KB, Klein TJ, Giurea A, Schumacher BL, Sah RL (2005). Inhibition of integrative cartilage repair by proteoglycan 4 in synovial fluid.. Arthritis Rheum.

[21] Funakoshi T, Schmid T, Hsu HP, Spector M (2008). Lubricin distribution in the goat infraspinatus tendon: A basis for interfascicular lubrication.. J Bone Joint Surg Am.

[22] Sun Y, Berger EJ, Zhao C, Jay GD, An KN, Amadio PC (2006). Expression and mapping of lubricin in canine flexor tendon.. J Orthop Res.

[23] Sun Y, Berger EJ, Zhao CF, An KN, Amadio PC, Jay G (2006). Mapping lubricin in canine musculoskeletal tissues.. Connect Tissue Res.

[24] Shine KM, Spector M (2008). The presence and distribution of lubricin in the caprine intervertebral disc.. J Orthop Res.

[25] Jay GD, Harris DA, Cha CJ (2001). Boundary lubrication by lubricin is mediated by O-linked beta(1–3)Gal-GalNAc oligosaccharides.. Glycoconj J.

[26] Elsaid KA, Jay GD, Warman ML, Rhee DK, Chichester CO (2005). Association of articular cartilage degradation and loss of boundary-lubricating ability of synovial fluid following injury and inflammatory arthritis.. Arthritis Rheum.

[27] Schaefer DB, Wendt D, Moretti M, Jakob M, Jay GD, Heberer M, Martin I (2004). Lubricin reduces cartilage-cartilage integration.. Biorheology.

[28] Klein J (2006). Molecular mechanisms of synovial joint lubrication.. Proc Inst Mech Eng, Part J: J Eng Tribol.

[29] Chang DP, Abu-Lail NI, Guilak F, Jay GD, Zauscher S (2008). Conformational mechanics, adsorption, and normal force interactions of lubricin and hyaluronic acid on model surfaces.. Langmuir.

[30] Radin EL, Swann DA, Weisser PA (1970). Separation of a Hyaluronate-Free Lubricating Fraction from Synovial Fluid.. Nature.

[31] Swann DA, Sotman S, Dixon M, Brooks C (1977). Isolation and Partial Characterization of Major Glycoprotein (Lgp-I) from Articular Lubricating Fraction from Bovine Synovial-Fluid.. Biochem J.

[32] Swann DA, Slayter HS, Silver FH (1981). The molecular structure of lubricating glycoprotein-I, the boundary lubricant for articular cartilage.. J Biol Chem.

[33] Jay GD, Lane BP, Sokoloff L (1992). Characterization of a Bovine Synovial-Fluid Lubricating Factor. 3. The Interaction with Hyaluronic-Acid.. Connect Tissue Res.

[34] Zappone B, Ruths M, Greene GW, Jay GD, Israelachvili JN (2007). Adsorption, lubrication, and wear of lubricin on model surfaces: Polymer brush-like behavior of a glycoprotein.. Biophys J.

[35] Su JL, Schumacher BL, Lindley KM, Jakob M (2001). DSoloveychik V, Burkhart W, Triantafillou JA, Kuettner K, Schmid T. Detection of superficial zone protein in human and animal body fluids by cross-species monoclonal antibodies specific to superficial zone protein.. Hybridoma.

[36] Laemmli UK (1970). Cleavage of structural proteins during the assembly of the head of bacteriophage T4.. Nature.

[37] Tsai PS, Evans JE, Green KM, Jakob M, Sullivan RM, Schaumberg DA, Richards SM, Dana MR, Sullivan DA (2006). Proteomic analysis of human meibomian gland secretions.. Br J Ophthalmol.

[38] Moghani T, Butler JP, Loring SH (2009). Determinants of friction in soft elastohydrodynamic lubrication.. J Biomech.

[39] Zhu H, Chauhan A (2007). A mathematical model of tear mixing under the lower lid.. Curr Eye Res.

[40] Tong L, Zhou L, Beuerman RW, Zhao SZ, Li XR (2011). Association of tear proteins with Meibomian gland disease and dry eye symptoms.. Br J Ophthalmol.

[41] Arita R, Itoh K, Inoue K, Kuchiba A, Yamaguchi T, Amano S (2009). Contact lens wear is associated with decrease of meibomian glands.. Ophthalmology.

